# Mechanical Performance of Asphalt Mortar Containing Hydrated Lime and EAFSS at Low and High Temperatures

**DOI:** 10.3390/ma10070743

**Published:** 2017-07-03

**Authors:** Ki Hoon Moon, Augusto Cannone Falchetto, Di Wang, Chiara Riccardi, Michael P. Wistuba

**Affiliations:** 1 Pavement Research Division, Korea Expressway Corporation (KEC), Hwa Sung city, Gyung Gi do 18489, Korea; moonx113@umn.edu; 2 Department of Civil Engineering, Braunschweig Pavement Engineering Centre—ISBS, Technical University of Braunschweig, Braunschweig 38106, Germany; di.wang@tu-bs.de (D.W.); chiara.riccardi@tu-braunschweig.de (C.R.); m.wistuba@tu-bs.de (M.P.W.)

**Keywords:** hydrated lime, Electric Arc-Furnace Steel Slag (EAFSS), asphalt mortar, Bending Beam Rheometer (BBR), Dynamic Shear Rheometer (DSR), Huet model

## Abstract

In this paper, the possibility of improving the global response of asphalt materials for pavement applications through the use of hydrated lime and Electric Arc-Furnace Steel Slag (EAFSS) was investigated. For this purpose, a set of asphalt mortars was prepared by mixing two different asphalt binders with fine granite aggregate together with hydrated lime or EAFSS at three different percentages. Bending Beam Rheometer (BBR) creep tests and Dynamic Shear Rheometer (DSR) complex modulus tests were performed to evaluate the material response both at low and high temperature. Then, the rheological Huet model was fitted to the BBR creep results for estimating the impact of filler content on the model parameters. It was found that an addition of hydrated lime and EAFSS up to 10% and 5%, respectively, results in satisfactory low-temperature performance with a substantial improvement of the high-temperature behavior.

## 1. Introduction

Low-temperature cracking represents one of the major distresses for asphalt pavement built in cold regions such as northern U.S. and northern Europe [1,2]. As temperature decreases, a considerable increase in thermal stress is observed up to the material strength limit, beyond which cracks start developing on the pavement surface. Other phenomena, such as traffic loading and freeze-thaw cycles can further accelerate the structural deterioration eventually leading to premature failure [3]. A simple solution to this distress is given by the application of softer asphalt binders, with considerably low Performance Grade (PG) [4] and better relaxation capabilities. However, this may potentially compromise the high-temperature response of the pavement inducing undesirable rutting phenomena. This is especially true for geographical location where extreme temperature variations are experienced between summer and winter, for which even a wide PG range cannot fully guarantee satisfactory pavement response.

The use of reinforcement materials, such as fine particles and fillers, represents an alternative solution to improve the rutting resistance when the low-temperature properties are not significantly affected by a potential increase in brittleness. A number of studies investigated how materials such as silica fume and hydrated lime affect the properties of asphalt materials from a mechanical and rheological viewpoint [5,6,7,8]. When these types of filler or fine particles are combined with asphalt binder, asphalt mastic or asphalt mortar are obtained. These materials have arisen significant interest in the recent past since their behavior is much closer to that of asphalt mixture than plain or modified asphalt binder [6,8,9,10]. In addition, they do not require very expensive testing devices, such servo-hydraulic machines, and the standard testing equipment for asphalt binder characterization can be easily used to determine their mechanical properties [9,10,11,12].

Many researchers investigated the possibility of relating the properties of asphalt mastic and mortar to the corresponding asphalt mixture behavior showing that type, size, grading and particles concentration significantly affect the final performance [9,10,11,12,13,14]. In most cases, an improvement in rutting, fatigue cracking, and moisture damage resistance was observed coupled to an overall stiffening effect [10,11,15,16,17]. For example, the research conducted at the École Nationale des Travaux Publics de l’État (ENTPE) in France, demonstrated that when ultra-fine particles are incorporated with asphalt binder, a considerable stiffening effect is observed especially at higher temperature [7,14,15]. Such an increase in stiffness is associated with obvious practical benefits in terms of resistance to permanent deformation and may appear as a simple and economical solution to mitigate (and/or reduce) rutting phenomena for road authorities and department of transportations.

However, not much attention was devoted to the effect of the inclusions of finer particles at low temperature. In most of the cases the response of mastic and mortar was only addressed in terms of complex modulus, and hence stiffness, while, the response in terms of relaxation properties, the evolution of thermal stress and the associated critical cracking temperature were not evaluated together with the high temperature performance [9,10,11,12,13,14,15,16,17]. Among the different reinforcement materials, Electric Arc-Furnace Steel Slag (EAFSS) represent a valuable option given the large amount available coupled to the environmental benefits associated with the recycling process of this industrial by-product [18,19,20,21,22].

## 2. Literature Review

Slag from the iron and steel industry are obtained from the rapid cooling process of the oxidized and superficial liquid phase present in electric arc furnaces, from circa 1300 °C to room temperature [18,19]. EAFSS are mainly composed of calcium, iron, aluminum, magnesium and silicon oxides, which together account for an average of approx. 90% of the weight of the material [20,21]. The principal component is iron, the presence of which is due to the action of the oxidative regime of the electric arc furnace on the liquid metal bath.

A number of studies were performed in the past [22] and more recently [23,24] to address the use of EAFSS in asphalt mixture, although not many have been devoted to the use of the fine fraction of this material. Sofilic et al. [21] evaluated the feasibility of using EAFSS as an alternative aggregate source in asphalt pavement by performing several microstructural analyses. It was found that EAFSS does not contain environmentally harmful elements and that they can be used as alternative aggregate for asphalt pavement construction and surface treatments. Skid resistance, permeability, porosity and surface texture of asphalt mixture prepared with EAFSS was evaluated by Liapis and Likoydis [25]. Based on field testing of highway sections, it was observed that EAFSS mixtures present performance comparable to the conventional paving mixtures. In the recent past, Mahmoud et al. [26] evaluated the possibility of using EAFSS in combination with the Warm Mix Asphalt (WMA) technology in substitution of natural limestone aggregate. Scanning Electron Microscope analysis and mechanical testing showed that, in spite of higher porosity and roughness, EAFSS mixture presented enhanced Marshall stability, resilient modulus, tensile strength, together with reduced moisture sensitivity and permanent deformation in comparison to conventional mixtures.

The use of alternative steel slags was also investigated over the years. This includes blast furnace granulated slag [27] and Linz-Donawitz (LD) slag. The latter were used for partially or entirely replacing the conventional aggregate skeleton [28]. The experimental results indicated that asphalt mixtures prepared with slag are suitable for asphalt pavement construction and that, in most cases, they perform better than conventional asphalt mixtures designed with natural aggregates.

## 3. Objective and Research Approach

In this research, the effect of different types of filler on the low and high-temperature properties of asphalt mortar was investigated. For this purpose, two different asphalt binders, one aggregate type and two diverse filler types, hydrate lime and EAFSS were mixed together to prepare a set 12 asphalt mortars. Hydrated lime conventionally used as filler for asphalt mixture and EAFSS were combined with a conventional type of fine granite aggregate (<0.150 mm) at three different percentages: 5%, 10% and 20% (by volume).

Bending Beam Rheometer (BBR) (Cannon Instrument Company, State College, PA, USA) [29] and Dynamic Shear Rheometer (DSR) (Malvern Instruments Ltd, Malvern, UK) [30] tests were performed both on the original asphalt binders and on the corresponding mortars to evaluate the material response at low and high temperatures. Based on the experimental results, complex modulus, |G*(*ω*)|, creep stiffness, *S*(*t*), *m*-value, *m*(*t*), thermal stress, *σ*(*T*), and critical cracking temperature, *T_CR_*, were computed then graphically and statistically analyzed [31]. Specifically, two different computation approaches were used to determine the evolution of *σ*(*T*): the Hopkins and Hamming’s algorithm [32], which is a numerical solution to solve the convolution integral, and Park and Kims’ algorithm [33], which represents an advanced power law interconversion method. These two methods were selected as, in a different study, it was found that they provide the upper and lower limit boundaries for thermal stress and critical cracking temperature of asphalt mixtures [2].

**Figure 1:**
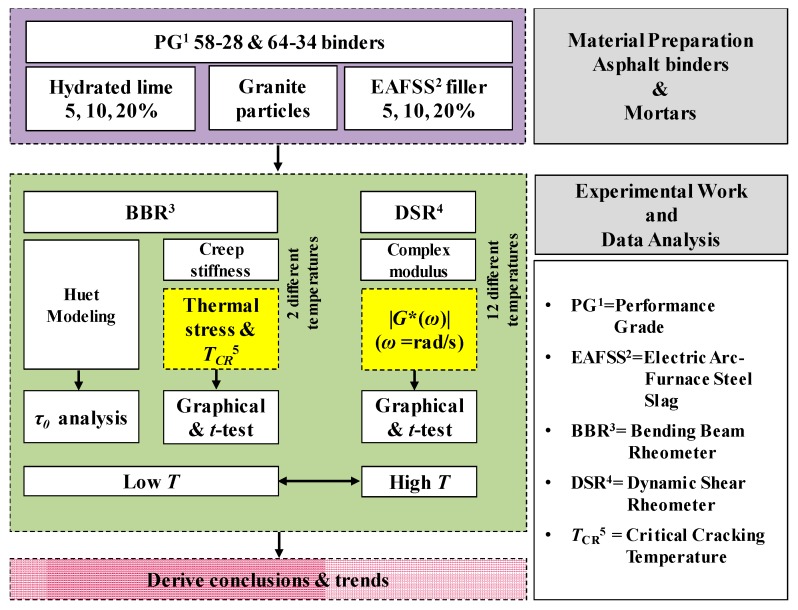
Research approach performed in this study.

Then, the rheological Huet model [34] was fitted to the low temperature creep stiffness data and a relation between the characteristic time, *τ*, of mortar and the filler content was obtained. In the case of DSR data analysis, Christensen Anderson and Marasteanu (CAM) model [35] and a simple sigmoidal function [36,37] were used for generating |G*(*ω*)| master curves which were then statistically compared to better understand the high temperature performance of the given asphalt materials. A schematic of the research approach selected for this study is presented in Figure 1.

## 4. Materials Testing

### 4.1. Materials Preparation

One plain PG 58-28 asphalt binder and one SBS polymer modified binder having PG 64-34 were prepared at the asphalt material laboratory of Korea Expressway Corporation (KEC). Both binders were combined with the fine portion (0.075 < *d* < 0.150 mm) of granite aggregate and mixed either with hydrated lime or EAFSS filler. The lower part of the sieve size curve of an asphalt mixture, AC11, commonly used for wearing course in Germany [38] was selected for establishing the relative proportions of fine granite particles and fillers. AC11 refers to a Hot Mix Asphalt (HMA) having nominal maximum aggregate size (NMAS) of 12.5 mm. Aggregates (fines and filler) were incorporated with the binder at three different percentages 5%, 10% and 20% (by volume), and a total of 12 mortars were prepared. The corresponding weight ratio according to volume fraction was 11.2%, 22.3% and 44.7%, and 16.5%, 32.9% and 65.8%, for hydrated lime and EAFSS, respectively, where the specific gravity of asphalt binder = 1.03 g/cm^3^, hydrated lime = 2.3 g/cm^3^ and EAFSS = 3.39 g/cm^3^ [39].

**Figure 2:**
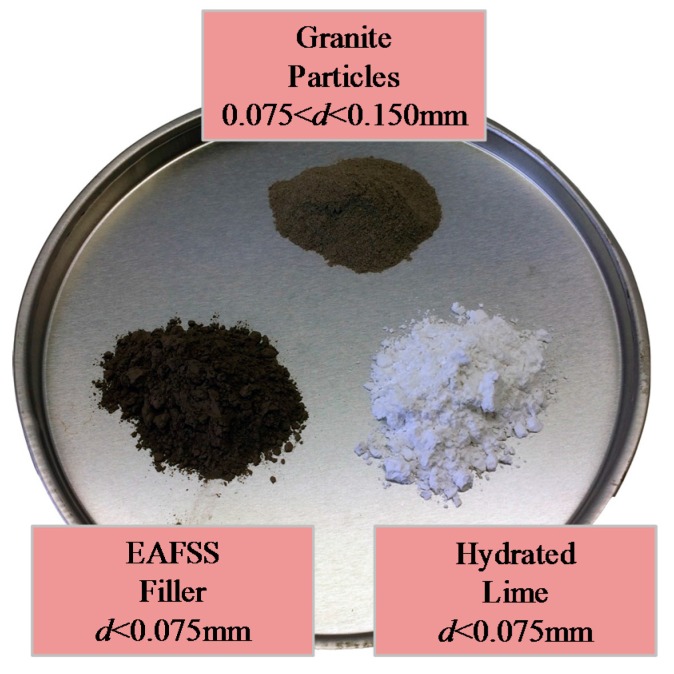
Granite, EAFSS, and hydrated lime particles.

In the case of Hot Mix Asphalt (HMA), averagely 5% asphalt binder (by weight) is used [1,3]. Therefore, approximately 0.6%, 1.1% and 2.2% of hydrated lime can be included (by weight) in the mix design when adding 5%, 10% and 20% based on binder volume, respectively. In the case of EAFSS, this corresponds to 0.8%, 1.6%, and 3.3%. A summary of the materials used in this research is presented in Table 1, while Figure 2 shows the three aggregate particles.

Asphalt binders, particles, and fillers were heated in the oven at the temperature range within 150 to 160 °C. Then, the filler was progressively added to the asphalt binder and the material was continuously mixed with a stirring device in order to achieve a homogenous distribution and to avoid the formation of lumps. Asphalt binders and corresponding mortars were short and long term aged with the Rolling Thin-Film Oven Test (RTFOT) (James Cox & Sons, Inc., Colfax, CA, USA) [40] and the Pressurized Aging Vessel (PAV) (Prentex Alloy Fabricators, Inc., Dallas, TX, USA) [41], and then, tested with the DSR [30] and the BBR [29] at the corresponding aging conditions (RTFOT and PAV aged binder for DSR and BBR), respectively.

### 4.2. Low-Temperature Testing and Parameters Computation

BBR creep tests (Cannon Instrument Company, State College, PA, USA) [29] were performed to determine the low-temperature properties of binder and corresponding mortar. In this test method, a constant load of 980 mN is applied for 240 s on small asphalt binder and mortar beams (*l* = 102 mm, *b* = 12.5 mm, *h* = 6.25) in three-point bending (3PB) configuration. Two different temperature, *low*PG + 10 °C and *low*(PG + 10) − 6 °C, were considered in order to generate relaxation modulus, *E*(*t*), master curves, thermal stress, *σ*(*T*), and corresponding critical cracking temperature, *T_CR_*. Three asphalt binder and mastic replicates were tested at each temperature. Both binders and mortars were subjected to long-term aging before testing [41]. Based on the BBR test creep stiffness, *S*(*t*), and *m*-value, *m*(*t*), were computed according to the following well-known formulas [1,2,3,29]:

(1) $$S\left(t\right)=\frac{1}{D\left(t\right)}=\frac{\sigma }{\varepsilon \left(t\right)}=\frac{P\cdot l^{3}}{4\cdot b\cdot h^{3}\cdot \delta \left(t\right)},$$

(2) $$m\left(t\right)=\left|\frac{d\mathrm{Log}\;S\left(t\right)}{d\mathrm{Log}\;t\;}\right|$$

where *S*(*t*) is the time-dependent flexural creep stiffness (MPa), *D*(*t*) is the creep compliance (1/MPa) = 1/*S*(*t*), *σ*(*t*) is the bending stress in the beam (MPa), *ε*(*t*) is the time-dependent bending strain in the beam (mm/mm), *P* is the applied constant load (=980 ± 50 mN), *δ*(*t*) is the beam deflection (mm), *l*, *b*, *h* are the beam dimensions (mm), *t* is time (s), respectively. 

In order to compute thermal stress, *σ*(*T*), *S*(*t*) was first numerically converted to the corresponding relaxation modulus, *E*(*t*), based on two different computation approaches: the Hopkins and Hamming’s algorithm [2,32] and Park and Kims’ power law interconversion method [2,33]. The Hopkins and Hamming algorithm [32] computes *E*(*t*) from *D*(*t*) by numerically solving the convolution integral [42]; see Equations (3)–(6). On the other hand, the Park and Kim’s approach (Equations (7) and (8)) estimates *E*(*t*) using a power law expression, which is a function of the rescaled time, t* [33]. Based on the findings obtained in a previous research [2], these two solutions were used in the present study to provide a more extensive data analysis of the experimental results.

(3) $$t=\overset{t}{\underset{0}{\int }}E\left(\tau \right)\cdot D\left(t-\tau \right)d\tau =\overset{t}{\underset{0}{\int }}E\left(t-\tau \right)\cdot D\left(\tau \right)d\tau ,$$

(4) $$t_{n+1}=\overset{t_{n}+1}{\underset{0}{\int }}E\left(\tau \right)\cdot D\left(t_{n+1}-\tau \right)d\tau =\overset{n}{\underset{i=0}{\sum }}\overset{t_{i}+1}{\underset{t_{i}}{\int }}E\left(\tau \right)\cdot D\left(t_{n+1}-\tau \right)d\tau ,$$

(5) $$\overset{t_{i}+1}{\underset{t_{i}}{\int }}E\left(\tau \right)\cdot D\left(t_{n+1}-\tau \right)d\tau =-E\left(t_{i+\frac{1}{2}}\right)\cdot \left[f\left(t_{n+1}-t_{i+1}\right)-f\left(t_{n+1}-t_{i}\right)\right],$$

(6) $$E\left(t_{n+\frac{1}{2}}\right)=\frac{t_{n+1}-\sum_{i=0}^{n-1}E\left(t_{i+\frac{1}{2}}\right)\cdot \left[f\left(t_{n+1}-t_{i}\right)-f\left(t_{n+1}-t_{i+1}\right)\right]}{f\left(t_{n+1}-t_{n}\right)}\;=\frac{t_{n+1}-\sum_{i=0}^{n-1}E\left(t_{i+\frac{1}{2}}\right)\cdot \left[f\left(t_{i+1}-t_{i}\right)\right]}{f\left(t_{n+1}-t_{n}\right)},$$

where

$$t_{i+1/2}=\frac{1}{2}\left(t_{i+1}+t_{i}\right),\;f\left(t\right)=\int_{0}^{t}D\left(t\right)dt,\;f\left(t_{0}\right)=0,\;E\left(t_{0}\right)=0,\;E\left(t_{1}\right)=t_{1}/f\left(t_{1}\right).$$

(7) $$1=E\left(t^{*}\right)\cdot D\left(t\right)=E\left({\left[\frac{\sin\left(n\cdot \pi \right)}{n\cdot \pi }\right]}^{\frac{1}{n}}\cdot t\right)\cdot D\left(t\right)\;\;\;\;\to E\left(t\right)=\frac{1}{D\left(t/{\left[\frac{\sin\left(n\cdot \pi \right)}{n\cdot \pi }\right]}^{\frac{1}{n}}\right)},$$

(8) $$n={\left|\frac{dLogD\left(t\right)}{Log\tau }\right|}_{\tau =t}\;\mathrm{or}\;n={\left|\frac{dLogE\left(t\right)}{Log\tau }\right|}_{\tau =t}$$

Then, *E*(*t*) master curves were generated using the CAM model with simple power law shift factor relation (see Equations (9) and (10)) [35] based on the values of *E*(*t*) computed through Equations (6) and (7), respectively:

(9) $$E\left(t\right)=E_{g}\cdot {\left[1+{\left(\frac{t}{t_{c}}\right)}^{v}\right]}^{-w/v}.\;\to LogE\left(t\right)=LogE_{g}+\left(-\frac{w}{v}\right)\cdot Log\left[1+{\left(\frac{t}{t_{c}}\right)}^{v}\right]\;\to LogE\left(t\right)=\mathrm{Log}\left(E_{g}=3\mathrm{GPa}\right)+\left(-\frac{w}{v}\right)\cdot Log\left[1+{\left(10^{Logt-Logt_{c}}\right)}^{v}\right]$$

(10) $$a_{T}=10^{C_{1}+C_{2}\cdot T_{S}}\;\;\to Loga_{T}=C_{1}+C_{2}\cdot T_{S}$$

where *E_g_* is the glassy modulus (assumed as 3 GPa for binders: [1]), *t_c_*, *ν*, *w* are fitting parameters (i.e., constants), *C*_1_, *C*_2_ are fitting parameters (i.e., constants), *T_S_* is reference temperature (*T_S_* = *low*PG + 10 °C; −18 °C and −24 °C for PG 58-28, PG 64-34 binder, respectively).

Finally, thermal stress was computed based on Equation (11); the one-dimensional hereditary integral, was solved with the 24 Gaussian points quadrature, assuming a cooling rate of 2 °C/h within a temperature range between 20 °C and −40 °C as [1,2]:

(11) $$\sigma \left(\xi \right)=\overset{\xi }{\underset{-\infty }{\int }}\frac{d\varepsilon \left(\xi \prime \right)}{d\xi \prime }\cdot E\left(\xi -\xi \prime \right)d\xi \prime =\overset{t}{\underset{-\infty }{\int }}\frac{d\left(\alpha \Delta T\right)}{dt\prime }\cdot E\left(\xi \left(t\right)-\xi \prime \left(t\right)\right)dt\prime ,$$

where ε(ξ′) is the strain rate, $\xi =t/a_{T}$ is the reduced time, *α* is the coefficient of thermal contraction of asphalt binder assumed to be equal to 0.00017/°C [1].

In addition, the critical cracking temperature, *T_CR_*, was computed from the results of thermal stress of the given asphalt binders and asphalt mortars based on the Single Asymptote Procedure (SAP) method [1,43], since no strength tests were conducted for this research. The experimentally obtained *σ*(*T*) and *T_CR_* of each two sets of binder and mortars from Equations (6), (7), (9)–(11) were further evaluated based on statistical analysis and rheological modeling as later described in the present paper.

### 4.3. High-Temperature Testing

DSR asphalt binder tests [30] were performed to measure complex modulus, |G*(*ω*)|, and phase angle, *δ*, and to determine the conventional |G*(*ω*)|/sin*δ* parameter commonly used for obtaining the binder PG [4] with respect to rutting resistance. This parameter has received a number of criticisms over the years as it is unable to fully capture the response of modified binders and to well correlate to rutting measurements, leading to the development of an alternative standard for determining the binder PG [44]. Nevertheless, in the present study, this parameter was selected since it provides a simple and practical approach to the evaluation of the material response to permanent deformations.

Tests were conducted with a plate-plate geometry having an 8mm diameter and 2 mm gap for temperatures between 4 and 40 °C and with a 25 mm diameter and 1 mm gap in the temperature range 28 °C to 70 °C. A temperature step of 6 °C was used to cover the entire temperature window, while the following angular frequencies (*ω*) were imposed to the specimens: 1.000, 1.586, 2.512, 3.980, 6.309, 10.000, 15.840, 25.120, 39.810, 63.090, and 100.000 rad/s. Similar to the BBR creep tests, three replicates were prepared and tested. All DSR tests were conducted on short term aged binders and mortars [40]. CAM model [35] and the sigmoidal function proposed by Pellinen [36] were used for generating |G*(*ω*)| master curves according to Equations (12) and (13), respectively, and assuming a reference temperature, *T_S_* = 22 °C. The expression of the CAM model for the complex modulus is:

(12) $$\begin{array}{cc} \left|G^{*}\left(\omega \right)\right| & =\left|G_{\infty }^{*}\left(\omega \right)\right|\cdot {\left[1+{\left(\frac{\omega }{t_{c}}\right)}^{\mu }\right]}^{-\frac{\beta }{\mu }}\\ \to \mathrm{Log}\left|G^{*}\left(\omega \right)\right| & =Log\left|G_{\infty }^{*}\left(\omega \right)\right|+\left(-\frac{\beta }{\mu }\right)\cdot Log\left[1+{\left(\frac{\omega }{t_{c}}\right)}^{\mu }\right]\\ & =Log\left|G_{\infty }^{*}\left(\omega \right)\right|+\left(-\frac{\beta }{\mu }\right)\cdot Log\left[1+{\left(10^{Log\omega -Logt_{c}}\right)}^{\mu }\right]\\ & =Log\left|\frac{E_{g}=3\mathrm{GPa}}{2\cdot \left(1+\nu \right)}\right|+\left(-\frac{\beta }{\mu }\right)\cdot Log\left[1+{\left(10^{Log\omega -Logt_{c}}\right)}^{\mu }\right] \end{array}$$

while the sigmoidal model can be expressed as:

(13) $$\begin{array}{cc} Log\left|G^{*}\left(\omega \right)\right| & =\delta +\frac{\alpha }{1+e^{\beta_{1}+\gamma \cdot Log\xi }}=\delta +\frac{\alpha }{1+e^{\beta_{1}+\gamma \cdot \left(Log\omega +Loga_{T}\right)}}\\ \to \left|G^{*}\left(\omega \right)\right| & =10^{\delta +\frac{\alpha }{1+e^{\beta_{1}+\gamma \cdot \left(Log\omega +Loga_{T}\right)}}} \end{array}$$

(14) $$\begin{array}{cc} E_{\infty } & =G^{*}\left(\omega \right)\cdot 2\cdot \left(1+\nu \right)\\ \to G^{*}\left(\omega \right) & =\frac{E}{2\cdot \left(1+\nu \right)} \end{array}$$

where $G_{\infty }^{*}\left(\omega \right)$ is the glassy modulus (assumed as 1.056~1.014 GPa, *v* = 0.42~0.48 for binders), *t_c_*, *μ* are fitting parameters (i.e., constants), *Log*a_T_ is the shift factor parameters (at 4, 10, 16, 28, 34, 40, 46, 52, 58, 64 and 70 °C). It should be remarked that the CAM model predicts relatively higher values of G*, while the simple sigmoidal approach provides G* values relatively lower with smoother S-shaped plot due to characteristics of the function.

## 5. Statistical Analysis: Methodology

The difference in *σ*(*T*), *T_CR_* and |G*(*ω*)| across the different asphalt binders and corresponding mortars was evaluated with a simple statistical *t*-test with 5% significance level (i.e., *α* = 0.05). To perform the analysis, the conditions of data normality and constant variance were assumed and formulated [31] while the testing hypotheses were expressed as:

(15) $$\mathrm{Null}\;\mathrm{hypothesis}:\;H_{0}:\mu_{A\left(\sigma ,\;T_{CR},\;G^{*}\right)}=\mu_{B\left(\sigma ,\;T_{CR},\;G^{*}\right)}$$

(16) $$\mathrm{Alternative}\;\mathrm{hypothesis}:\;H_{1}:\mu_{A\left(\sigma ,\;T_{CR},\;G^{*}\right)}\neq \mu_{B\left(\sigma ,\;T_{CR},\;G^{*}\right)}$$

where the mean (response) *μ* corresponds to *σ*(*T*), *T_CR_* and |G^*^(*ω*)| obtained from BBR and DSR tests. The pooled standard deviation, *S_P_*, can be computed as follows:

(17) $$S_{P\left(\sigma \left(T\right),\;T_{CR},\;G^{*}\right)}=\sqrt{\frac{\left(n_{A}\left(=3\right)-1\right)\cdot S_{A,\;\;\left(\sigma \left(T\right),\;T_{CR},\;G^{*}\right)}^{2}+\left(n_{B}\left(=3\right)-1\right)\cdot S_{B,\;\;\left(\sigma \left(T\right),\;T_{CR},\;G^{*}\right)}^{2}}{n_{A}\left(=3\right)+n_{B}\left(=3\right)-2}}$$

where $S_{A\left(\sigma \left(T\right),\;T_{CR},\;G^{*}\right)}$ is standard deviation of *σ*(*T*), *T_CR_* and |G^*^(*ω*)| (Group A), $S_{B\left(\sigma \left(T\right),\;T_{CR},\;G^{*}\right)}$ is standard deviation of *σ*(*T*), *T_CR_* and |G^*^(*ω*)| (Group B), *n_A_* and *n_B_* is number of specimens in Group A and B (*n* = 3), respectively.

By using Equation (18) the results of *t*-static can be computed as:

(18) $$t-static=\frac{\mu_{A\left(\sigma \left(T\right),\;T_{CR},\;G^{*}\right)}-\mu_{B\left(\sigma \left(T\right),\;T_{CR},\;G^{*}\right)}}{S_{P\left(\sigma \left(T\right),\;T_{CR},\;G^{*}\right)\cdot \sqrt{\frac{1}{n_{A}}+\frac{1}{n_{B}}}}}=\frac{\mu_{A\left(\sigma \left(T\right),\;T_{CR},\;G^{*}\right)}-\mu_{B\left(\sigma \left(T\right),\;T_{CR},\;G^{*}\right)}}{S_{P\left(\sigma \left(T\right),\;T_{CR},\;G^{*}\right)}\cdot \sqrt{\frac{2}{3}}}$$

where the degrees of freedom, $df=\left(n_{A}+n_{B}\right)-2=3+3-2+4$.

Based on Equation (18), the output of the statistical tests, *p*-value, can be computed and compared to the significant threshold (i.e., *α* = 0.05), to verify if the two compared groups can be considered statistically equivalent or different.

## 6. Low-Temperature Results and Modeling

In the present section, the experimental results and the statistical analysis of the low-temperature tests are first presented. Then, the Huet model [34], is fitted to the creep stiffness data, *S*(*t*), and the evolution the characteristic time, *τ*, which is a parameter governing the temperature dependency of the model and that is associated with the time needed for the system to relax, is further investigated.

### 6.1. Experimental Results and Statistical Analysis

**Figure 3:**
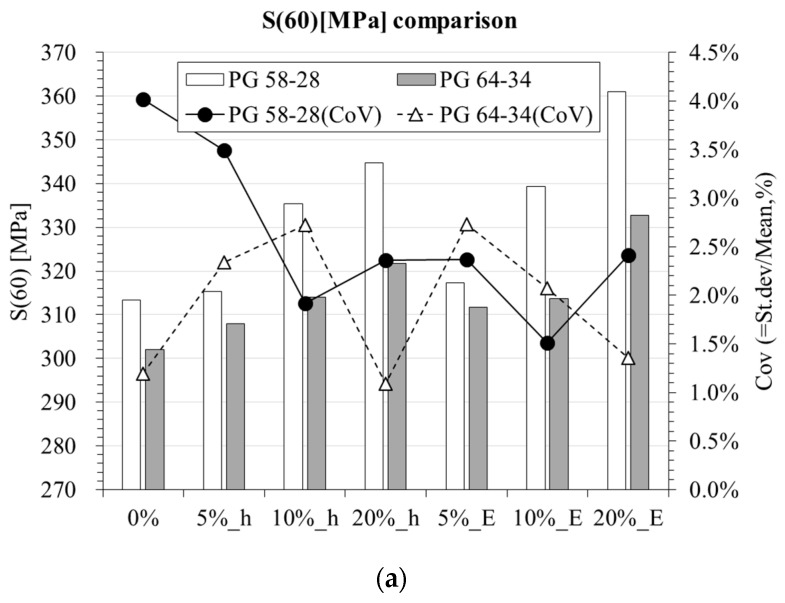

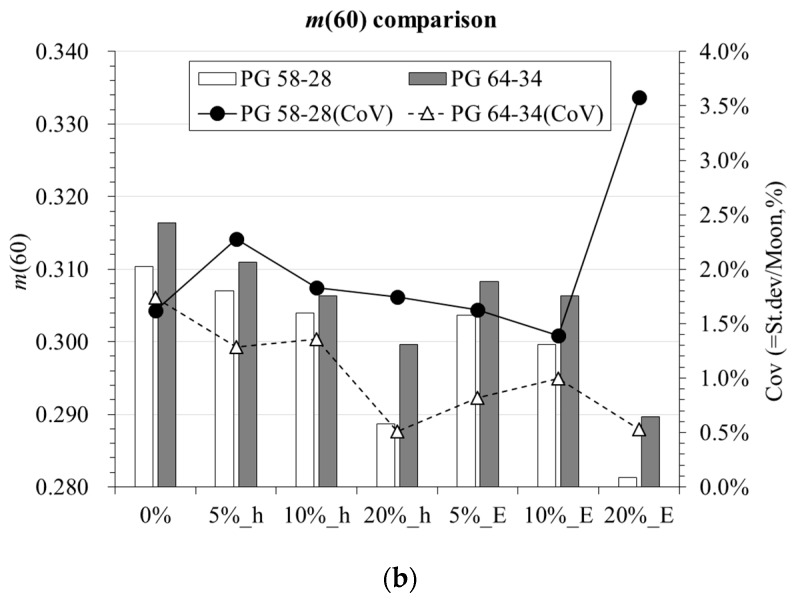
BBR results comparison. (**a**) *S*(60) [MPa] comparison; (**b**) *m*(60) comparison.

The experimentally measured creep stiffness and *m*-value at 60 s (see Equations (1) and (2)) are presented in Figure 3. A visual inspection of the two plots suggests that for low volume content of hydrated lime and EAFSS both creep stiffness and *m*-value of mortars are in the same order of magnitude of the original binder, while a significant increase of *S*(60 s) coupled to a considerable decrease of *m*(60 s) are observed when moving to higher particles concentration. However, it should be mentioned that up to 5% addition either of hydrated lime or EAFSS to the binder, no visual significant differences in *S*(60 s) and *m*(60 s) were observed. In addition, coefficients of variation (CoV) smaller than 5% provide evidence that BBR creep testing can be successfully used not only for asphalt binder but also for mortar specimens. It is also interesting to observe that although the two binders present *S*(60 s) close to the limiting stiffness value, they both exceed 300 MPa.

**Figure 4:**
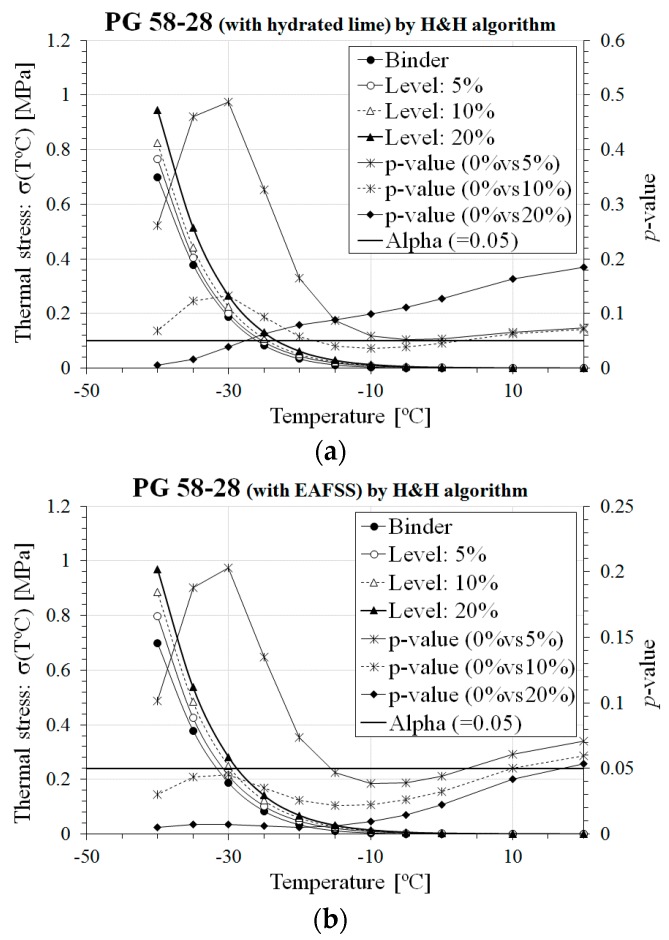

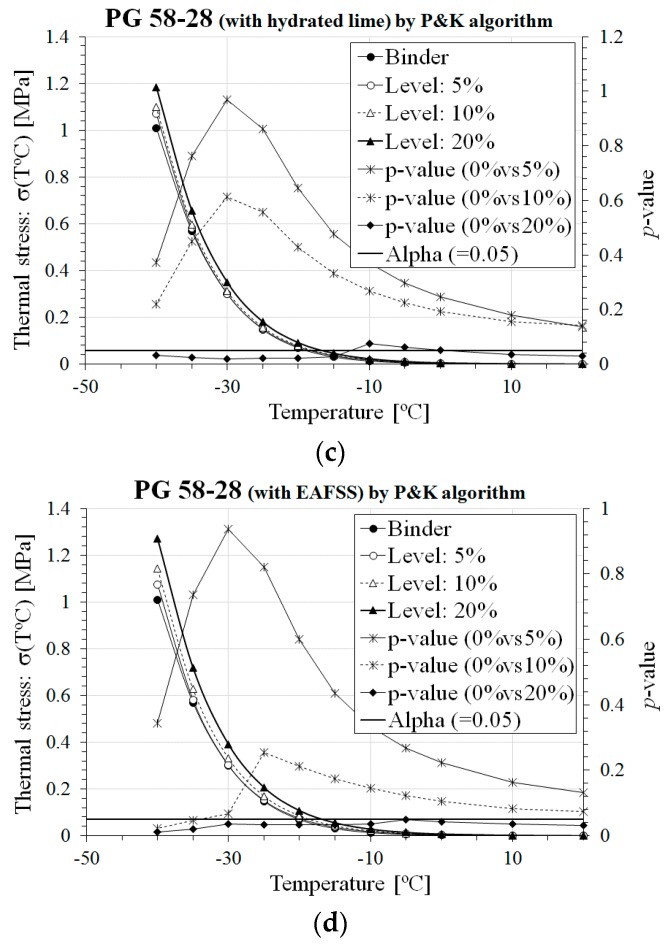
Comparison of *σ*(*T*) and *T_CR_* for PG 58-28 binder. (**a**) PG 58-28 (with hydrated lime) by H&H algorithm; (**b**) PG 58-28 (with EAFSS) by H&H algorithm; (**c**) PG 58-28 (with hydrated lime) by P&K algorithm; (**d**) PG 58-28 (with EAFSS) by P&K algorithm.

**Figure 5:**
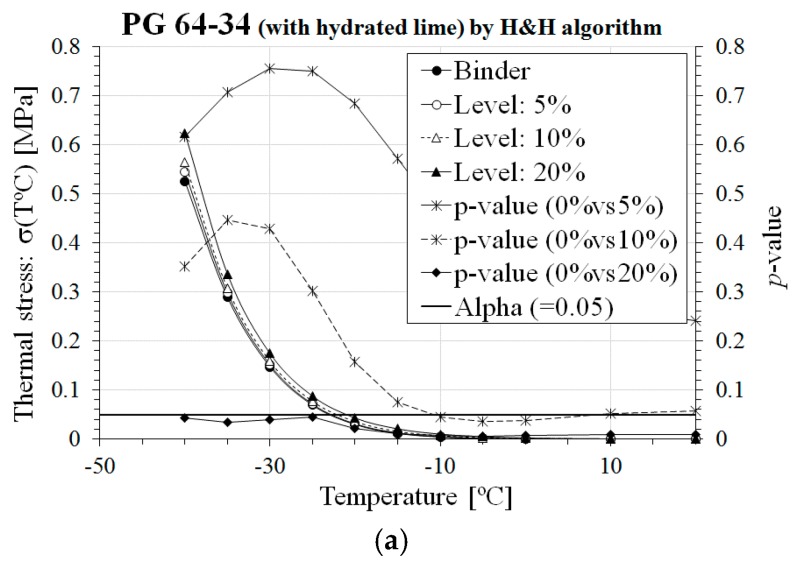

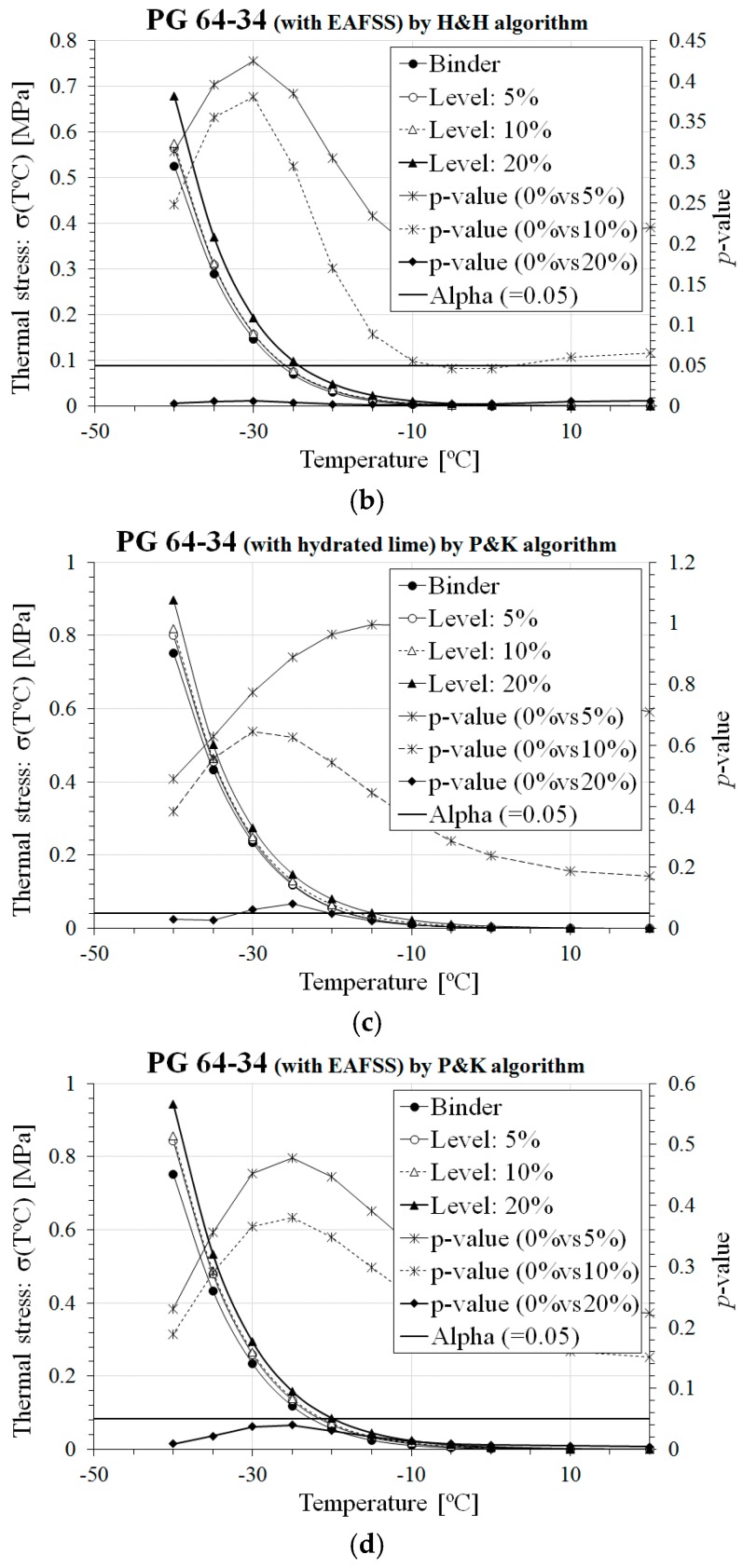
Comparison of *σ*(*T*) and *T_CR_* for PG 64-34 binder. (**a**) PG 64-34 (with hydrated lime) by H&H algorithm; (**b**) PG 64-34 (with EAFSS) by H&H algorithm; (**c**) PG 64-34 (with hydrated lime) by P&K algorithm; (**d**) PG 64-34 (with EAFSS) by P&K algorithm.

Based on Equations (1)–(11), thermal stress, *σ*(*T*), and critical cracking temperature, *T_CR_*, of asphalt binders and corresponding mortars were computed and then graphically and statistically compared based on *t*-test [31] with a 5% significance level (i.e., *α* = 0.05). The results and the statistical analysis are presented in Figure 4 and Figure 5 and Table 2, for *σ*(*T*) and *T_CR_*, respectively; bold values indicate the significance of the statistical comparison.

From the results of Table 2 and Figure 4 and Figure 5, an increase in thermal stress and *T_CR_* was obtained for higher content of hydrated lime and EAFSS with respect to the original asphalt binder for both *E*(*t*) computation approaches: Hopkins and Hammings [32] and Park and Kim [33]. This confirms what visually observed in the bar charts of Figure 3 for which poorer relaxation properties and higher stiffness characteristics were measured when the content of aggregate particles and filler was increased. It also should be noted that no significant differences in *σ*(*T*) and *T_CR_* were found for mortars prepared with the softer PG 64-34 binder and up to 10% of both hydrated lime and EAFSS filler. This is not the case for the stiffer PG 58-28 binder, where the EAFSS content has to be limited to 5%.

Overall, for low filler content, the low-temperature response of asphalt mortar is comparable with that of asphalt binder; this should guarantee satisfactory performance against thermal cracking. Nevertheless, with respect to filler type, the use of hydrated lime rather than EAFSS is advisable when combined with a stiffer binder to maintain a reasonable level of low temperature cracking resistance compared to non-filler added asphalt binder (see Figure 4 and Figure 5 and Table 2).

### 6.2. Huet Model and Analysis

**Figure 6:**
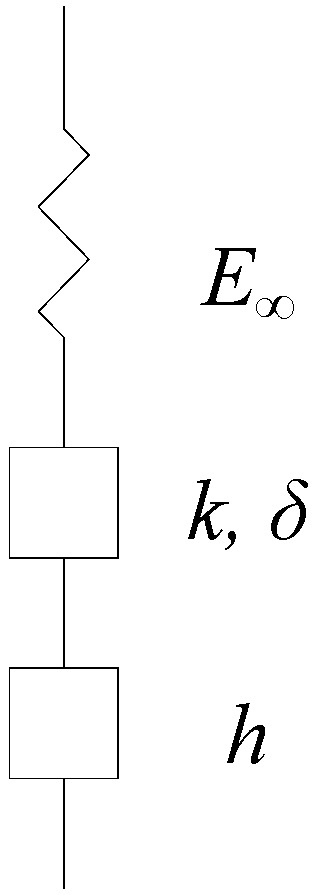
Huet model schematic [41].

In order to further evaluate the effect of particles contents on the material mechanical response at low temperature, the Huet model [34] was fitted to the experimental creep stiffness data. This model consists of two parabolic elements and a spring combined in series (Figure 6).

In the Huet model, the creep compliance, *D*(*t*), is expressed according to the following equation:

(19) $$D\left(t\right)=\frac{1}{S\left(t\right)}=\frac{1}{E_{\infty }}\cdot \left(1+\delta \frac{{\left(\frac{t}{\tau }\right)}^{k}}{Г\left(k+1\right)}+\frac{{\left(\frac{t}{\tau }\right)}^{h}}{Г\left(h+1\right)}\right),$$

where *S*(*t*) is creep stiffness(=1/*D*(*t*)), *E_∞_* is glassy modulus (=3 GPa, [1]), *h*, *k* is exponents such that 0 < *k* < *h* < 1, *δ* is dimensionless constant, *Γ* is gamma function.

(20) $$Г\left(n\right)=\int_{0}^{\infty }t^{n-1}\cdot e^{-t}dt,$$

(21) Г(n+1)=n·Г(n),

where *n* > 0 or Real (*n*) > 0, *t* is integration variable, *n* is argument of the gamma function.

(22) $$\tau =a_{T}\left(T\right)\cdot \tau_{0}\left(T_{s}\right),$$

where *τ* is the characteristic time, associated with the relaxation time of the material [12].

In previous studies [45,46], it was found that for viscoelastic materials such as asphalt binder, parameters *k* and *h* range within 0.08~0.3 and 0.3~0.8, respectively. In addition, it was also shown that stiffer materials are linked to lower values of *k* and *h* [34,46]. The five constants required by the Huet model (*δ*, *k*, *h*, *E_∞_*, and *τ*) were determined through the minimization of the sum of the distances between the experimental creep compliance and that predicted by the Huet model at *n* time points (see Equation (23)).

(23) $$\begin{array}{cc} \mathrm{Error} & =\overset{240\sec}{\underset{n=1}{\sum }}{\left[D{\left(t\right)}_{\mathrm{experimental}}-D{\left(t\right)}_{\mathrm{Heut}\;\mathrm{fitting}}\right]}^{2}\\ & =\overset{240\sec}{\underset{n=1}{\sum }}{\left[D{\left(t\right)}_{\mathrm{experimental}}-\frac{1}{E_{\infty }\left(=3\mathrm{GPa}\right)}\cdot \left(1+\delta \frac{{\left(t/\tau \right)}^{k}}{Г\left(k+1\right)}+\frac{{\left(t/\tau \right)}^{h}}{Г\left(h+1\right)}\right)\right]}^{2}≅0 \end{array}$$

**Figure 7:**
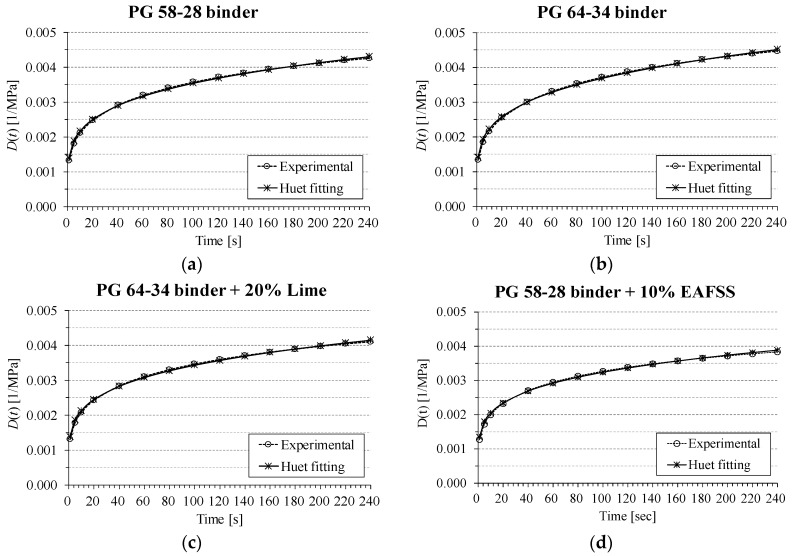
Huet model fitting of BBR binder and mortar (10 & 20%) *D*(*t*) = 1/*S*(*t*). (**a**) PG 58-28 binder; (**b**) PG 64-34 binder; (**c**) PG 64-34 binder + 20% Lime; (**d**) PG 58-28 binder + 10% EAFSS.

The fitting was limited to the low-temperature creep stiffens data obtained at lowPG + 10 °C, as the three kernel parameters of the model (*δ*, *k*, *h*) are the same over the entire spectrum of temperature as demonstrated in different research efforts [14,46]. Figure 7 provides examples of model fitting of both binder and mortar data.

With respect to the model parameters, the addition of fine aggregate particles was first investigated by evaluating the variation of parameters *k* and *h*, to obtain an indirect estimation of the stiffening effect. Table 3 and Table 4 present the values of *k* and *h* and the results of the statistical *t*-tests [31] conducted to determine any significant change in these two parameters depending on the content of hydrated lime and EAFSS; bold numbers indicate a significant difference in *k* and *h*.

A significant variation in *k* and *h* is observed (for both binders) for hydrated lime and EAFSS content above 10% and 5%, respectively. This suggests that EAFSS present a much more effective stiffening effect than what obtained with hydrated lime for the same volumetric content, substantially confirming the statistical trend observed for thermal stress and *T_CR_*. It must be stressed out that the difference in terms of binder PG is also confirmed, where the stiffer PG 58-28 seems to be more sensitive to the addition of EAFSS. In the case of softer asphalt binder (i.e., PG 64-34 binder), no significant difference at low temperature was observed up to 10% of hydrated lime and EAFSS.

**Figure 8:**
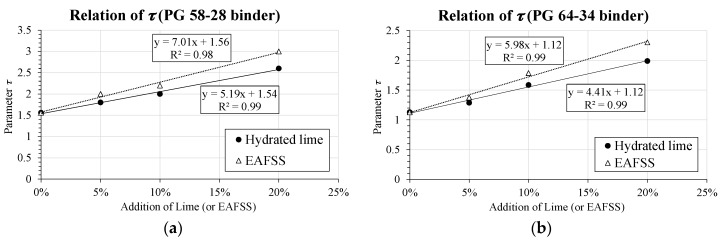
Relation between *τ* and aggregate particle content. (**a**) Relation of *τ* (PG 58-28 binder); (**b**) Relation of *τ* (PG 64-34 binder).

The effect of the particle content was also addressed by relating the percentage of material included in the mortar to the variation in characteristic time, *τ*. For example, if the value of *τ* is larger more time is required for the given material to relax at low temperature. As already mentioned, this parameter governs the temperature dependency of the model and provides information on the time required for the material to relax [12]. Figure 8 presents the relation obtained between *τ* and aggregate particle content for all the different materials used in the present study.

The two plots suggest that a clear proportional stiffening effect with a linear trend is linked to the addition of hydrated lime and EAFSS. Longer relaxation time is experienced for the EAFSS filler. This is true for both binder types (PG 58-28 and PG 64-34). In addition, substantially higher relaxation capabilities can be expected from materials prepared with PG 64-34 binder, confirming the results previously obtained. The comparison of the Huet model [34] parameter supports the trends observed in Section 6.1 of the present work.

## 7. High-Temperature Results and Analysis

In this section, the complex modulus, |G^*^(*ω*)|, obtained from DSR tests was graphically and statistically compared to evaluate the potential improvement in term of resistance to permanent deformations, which can be obtained at higher temperature condition when using hydrated lime and EAFSS. In order to perform a consistent comparison over a wider range of frequency, |G^*^(*ω*)| master curves were generated with the CAM model (Equation (12)) [35] and with a simple sigmoidal function (Equation (13)) [36].

**Figure 9:**
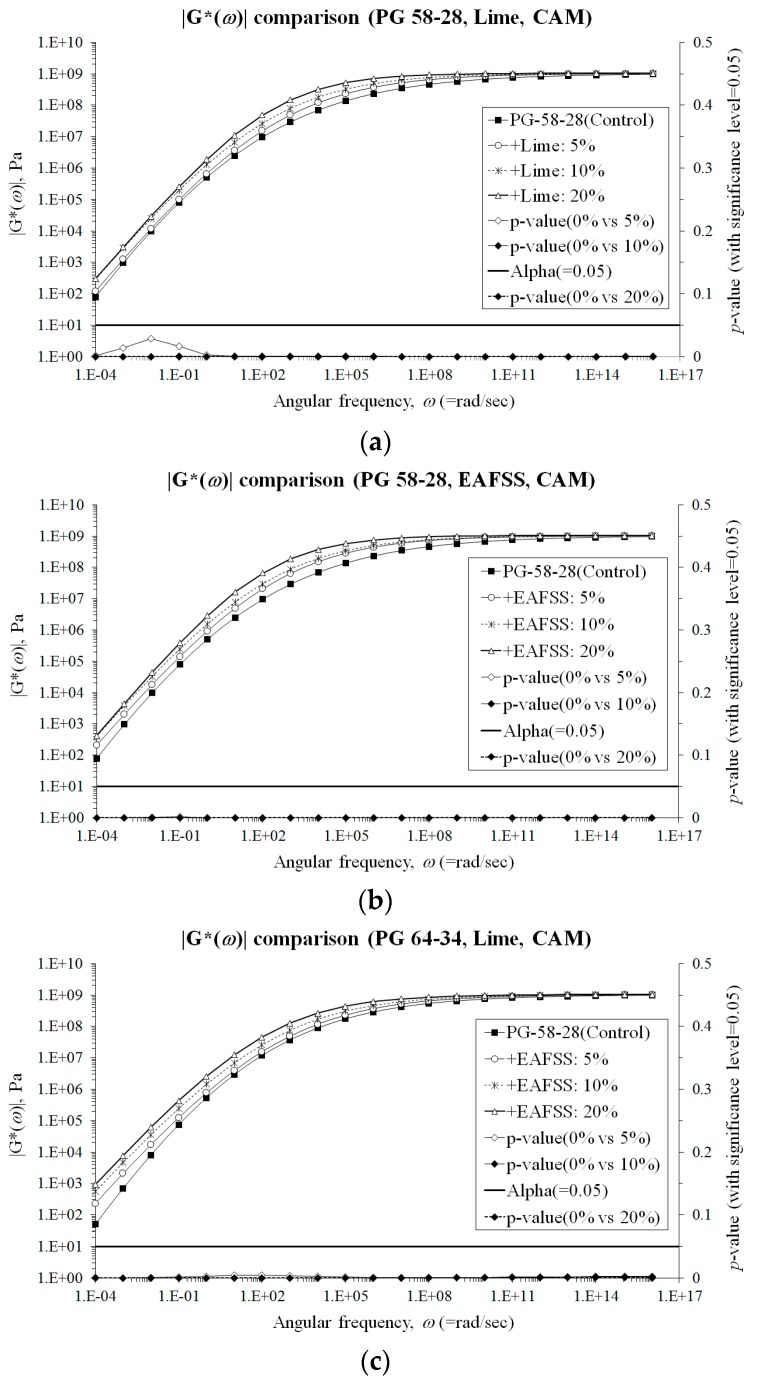

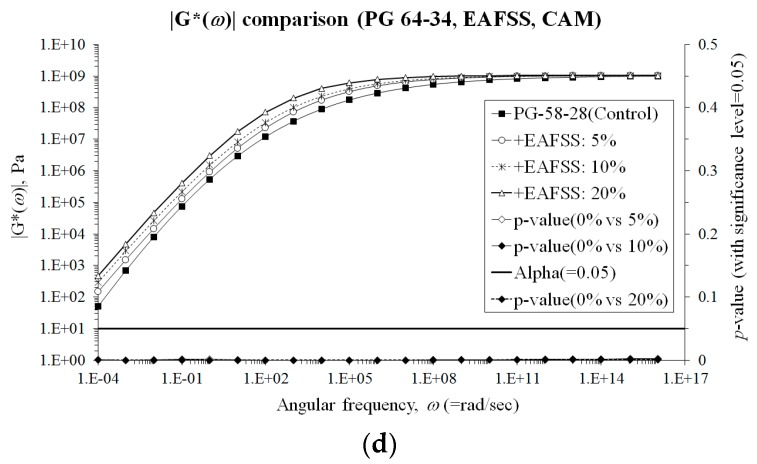
|G*(*ω*)| comparisons by using the CAM model [35]. (**a**) |G*(*ω*)| comparisons (PG 58-28, Lime, CAM); (**b**) |G*(*ω*)| comparisons (PG 58-28, EAFSS, CAM); (**c**) |G*(*ω*)| comparisons (PG 64-34, Lime, CAM); (**d**) |G*(*ω*)| comparisons (PG 64-34, EAFSS, CAM).

**Figure 10:**
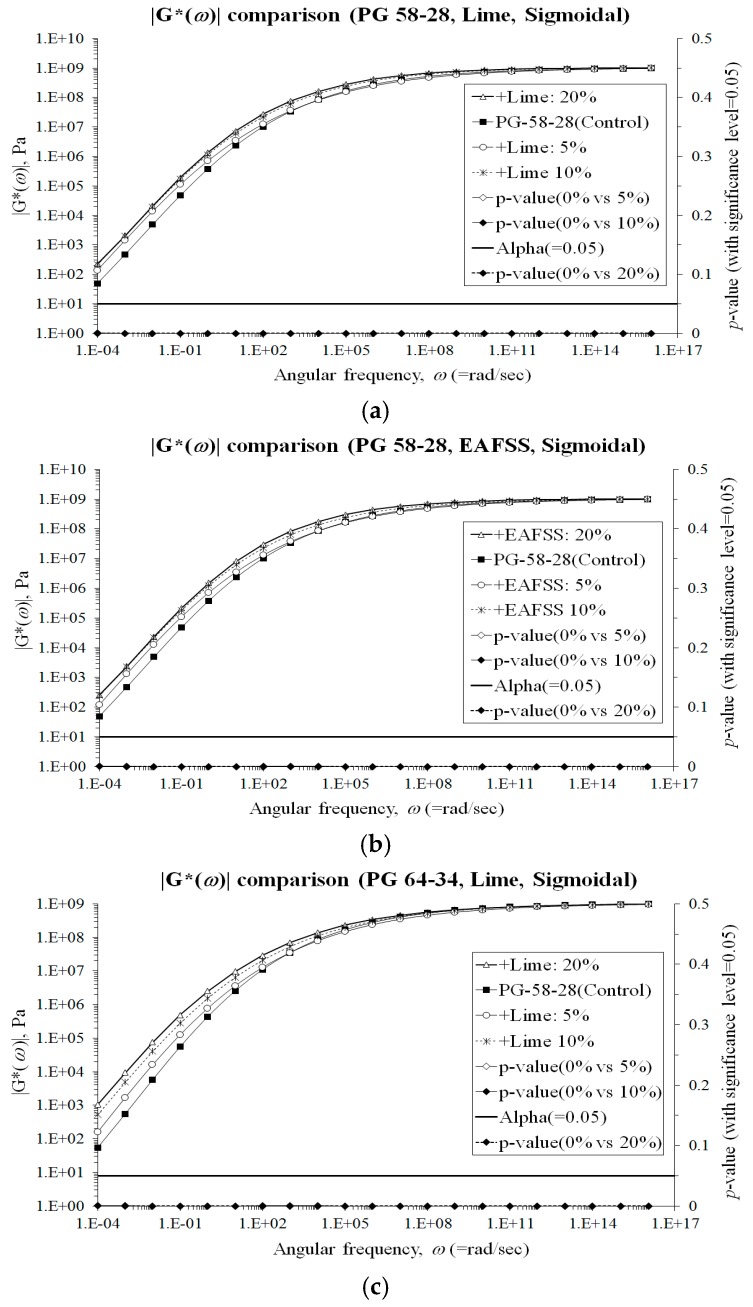

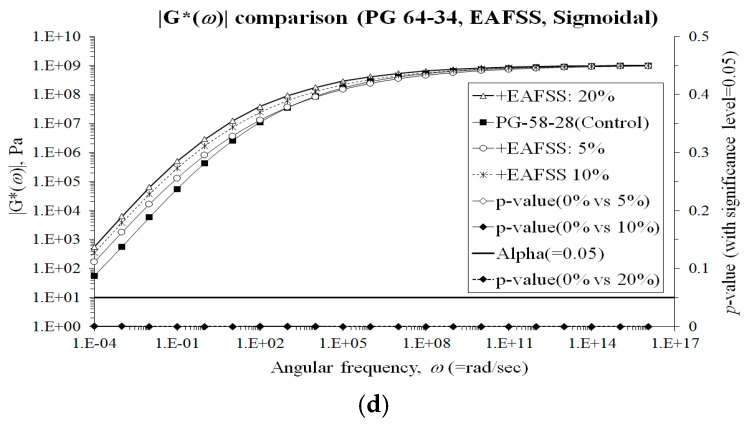
|G*(*ω*)| comparisons by using the sigmoidal function [45]. (**a**) |G*(*ω*)| comparisons (PG 58-28, Lime, Sigmoidal); (**b**) |G*(*ω*)| comparisons (PG 58-28, EAFSS, Sigmoidal); (**c**) |G*(*ω*)| comparisons (PG 64-34, Lime, Sigmoidal); (**d**) |G*(*ω*)| comparisons (PG 64-34, EAFSS, Sigmoidal).

Statistical *t*-tests (Equations (15)–(18)) was then conducted on the entire range of values for the different binders and mortars. The experimental and statistical results are shown in Figure 9 and Figure 10.

The master curves clearly show a significant increase in |G^*^(*ω*)| starting from the lower aggregate particles contents (5%). This is true for both binder types and for both hydrated lime and EAFSS. Therefore, better performance against rutting can be expected when adding 5% or more of hydrated lime and EAFSS. However, given the results of the low-temperature analysis (previous section), the filler content has to be limited to 10% and 5% for hydrated lime and EAFSS, respectively in order to avoid a substantial reduction in the corresponding relaxation properties and consistent increase in creep stiffness.

**Figure 11:**
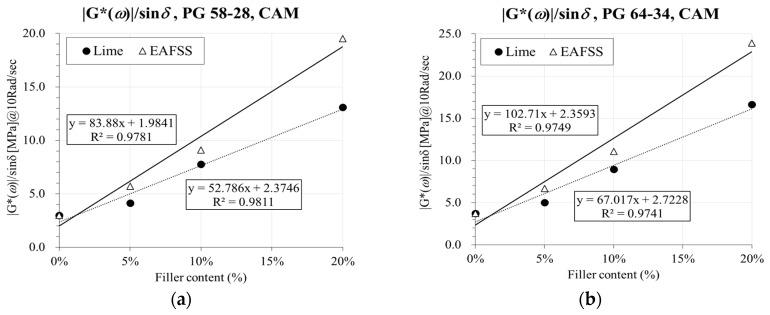

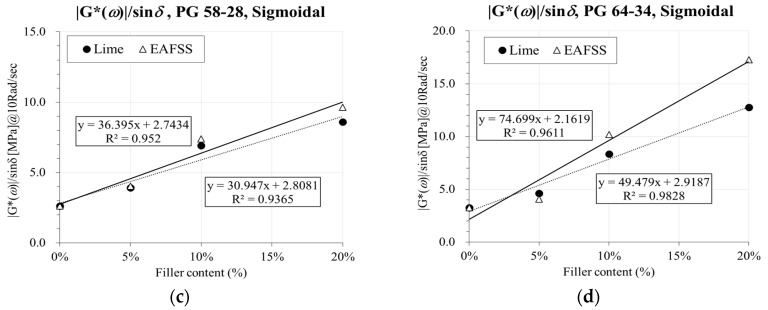
Relation between |G*(ω)|/sinδ and aggregate particle content with CAM [35] and simple Sigmoidal function [45]. (**a**) |G*(ω)|/sinδ, PG 58-28, CAM; (**b**) |G*(ω)|/sinδ, PG 64-34, CAM; (**c**) |G*(ω)|/sinδ, PG 58-28, Sigmoidal; (**d**) |G*(ω)|/sinδ, PG 64-34, Sigmoidal.

The conventional parameter |G^*^(*ω*)|/sin*δ* [4], was computed from the master curve at the corresponding shifting reference temperature (*T_S_* = 22 °C) and then used to further evaluate the stiffening effect with respect to rutting resistance. This parameter was then plotted against aggregate particle content as shown in Figure 11.

A reasonable linear trend is observed between |G^*^(*ω*)|/sin*δ* and the percentage of both hydrated lime or EAFSS. Higher particles content results in larger |G^*^(*ω*)|/sin*δ* ratios for both binders. As in the case of low-temperature creep stiffness, EAFSS induces a more consistent stiffening effect on the material. On the other hand, higher stiffening ratios are observed for PG 64-34 binder, showing an opposite trend to what measured at low temperature, where lower stiffness was obtained in comparison to the mortars prepared with PG 58-28 binder. It is hypothesized that the polymer modification affects the global stiffness of the binder at higher temperature leading to a more consistent structure when interacting with the reinforcement given by the aggregate inhomogeneities (i.e., particles).

Based on the results at low and high temperatures, the optimal aggregate particle content is limited to 10% for hydrated lime and EAFSS when using plain PG 64-34 binder. A slightly different result was found for mortars prepared with the modified PG 58-28 binder, for which 10% of hydrated lime, but only 5% EAFSS can be used without compromising the low-temperature performance.

## 8. Summary and Conclusions

In this paper, the possibility of using hydrated lime and EAFSS filler to improve the resistance of asphalt materials to permanent deformation without compromising the low-temperature performance was investigated. BBR creep and DSR complex modulus tests were conducted on two different binders and on 12 mortars prepared with fine granite particles and two type of fillers: hydrated lime and EAFSS. In addition, the Huet model was used to evaluate the effect of particles content on the model parameters. Based on the analysis performed, the following conclusions can be drawn:

- The low-temperature properties of mortars prepared with softer asphalt binder (PG 64-34 binder) are not significantly affected when using hydrated lime or EAFSS up to 10%. When a stiffer binder is used in the mix design, the EAFSS content has to be limited to 5%, suggesting a consistent stiffening effect for this type of filler.
- Higher material characteristic time was obtained from the Huet model for materials contenting higher amount of filler, suggesting a progressive loss in relaxation capabilities.
- At high temperature, the inclusion of hydrated lime and EAFSS results into a consistent improvement of the performance against rutting. This is shown by the increasing linear trend of the conventional PG rutting parameter for increased particles contents.

The use of hydrated lime and EAFSS filler is beneficial in terms of rutting resistance when the low-temperature characteristics of the original binder are not significantly affected. Given the sensitivity to smaller contents of EAFSS of the plain binder, hydrated lime is preferable from a practical viewpoint since large amounts can be incorporated in the mixture, potentially leaving room for mix design adjustments. Nevertheless, the possibility of incorporating EAFSS in the mix design represents a significant opportunity for recycling an industrial by-product with substantial environmental benefits.

The approach used in this research represents a simple practical tool for engineers when operating in regions experiencing climate with large yearly temperature range. Nevertheless, a larger testing campaign needs to be undertaken to provide additional support to methodology and results; this includes more binder and filler types, together with the use of alternative testing methods, such as the Multiple-Stress Creep-Recovery (MSCR) test. In addition, the present work highlight the need for identifying a set of robust limiting criteria for asphalt mortars as currently available for the selection of asphalt binder, based on the PG system [4] or as recently proposed for asphalt mixture at low temperature [47]. This is part of a joint research effort between Technical University of Braunschweig, Germany, and Korea Expressway Corporation (KEC), Research Division in South Korea.

## Figures and Tables

**Table 1:** Asphalt binders and mortars prepared in this study.

Binder Type	PG	Filler Type	Filler Content	Material	Reference
Plain	PG 58-28	Hydrated lime (*r* = 2.3 g/cm^3^)	0%	Binder	Control
			5%	Mortar	-
			10%	Mortar	-
			20%	Mortar	-
		EAFSS (*r* = 3.39 g/cm^3^)	0%	Binder	Control
			5%	Mortar	-
			10%	Mortar	-
			20%	Mortar	-
Modified	PG 64-34	Hydrated lime (*r* = 2.3 g/cm^3^)	0%	Binder	Control
			5%	Mortar	-
			10%	Mortar	-
			20%	Mortar	-
		EAFSS (*r* = 3.39 g/cm^3^)	0%	Binder	Control
			5%	Mortar	-
			10%	Mortar	-
			20%	Mortar	-

**Table 2:** *T_CR_* comparison results.

Variables	Particle Content	^1^ H&H Algorithm [41]				^2^ P&K algorithm [42]			
		PG 58-28		PG 64-34		PG 58-28		PG 64-34	
		^3^ H.Lime	EAFSS	H.Lime	EAFSS	H.Lime	EAFSS	H.Lime	EAFSS
*T_CR_* (°C)	0%	−28.8		−32.9		−28.3		−32.2	
	5%	−28.7	−28.4	−32.5	−32.5	−28.2	−28.2	−31.9	−32.0
	10%	−28.3	−27.6	−32.2	−32.4	−28.1	−27.5	−31.8	−31.8
	20%	−26.9	−26.7	−31.8	−31.3	−26.7	−26.5	−31.6	−30.8
*p*-value	0 vs. 5%	0.869	0.067	0.059	0.051	0.760	0.588	0.318	0.502
	0 vs. 10%	0.068	**0.003**	0.076	0.052	0.426	**0.018**	0.291	0.077
	0 vs. 20%	**0.008**	**0.001**	**0.021**	**0.001**	**0.014**	**0.005**	**0.046**	**0.012**

^1^ H&H: Hopkins and Hammings; ^2^ P&K: Park and Kim; ^3^ H.Lime: hydrated lime.

**Table 3:** Huet Model Parameters *k*: statistical comparison for PG 58-28 and PG 64-34 binder.

Parameter		PG 58-28		PG 64-34	
		H.Lime	EAFSS	H.Lime	EAFSS
*k*	Level 0%	0.101		0.143	
	Level 5%	0.097	0.093	0.132	0.128
	Level 10%	0.093	0.089	0.127	0.126
	Level 20%	0.087	0.079	0.116	0.101
*p*-value	0% vs. 5%	0.306	0.064	0.160	0.068
	0% vs. 10%	0.098	**0.044**	0.055	0.051
	0% vs. 20%	**0.011**	**0.002**	**0.009**	**0.005**

**Table 4:** Huet Model Parameters *h*: statistical comparison for PG 58-28 and PG 64-34 binder.

Parameter		PG 58-28		PG 64-34	
		H.Lime	EAFSS	H.Lime	EAFSS
*h*	Level 0%	0.533		0.646	
	Level 5%	0.523	0.499	0.623	0.615
	Level 10%	0.504	0.484	0.615	0.594
	Level 20%	0.467	0.453	0.572	0.555
*p*-value	0% vs. 5%	0.509	0.073	0.125	0.055
	0% vs. 10%	0.091	**0.029**	0.052	0.005
	0% vs. 20%	**0.014**	**0.009**	**0.004**	**0.002**
